# Setting Characteristics, Solubility, Bioactivity and Interaction with Dentin of Four Calcium Silicate-Based Endodontic Sealers

**DOI:** 10.3390/jfb17040192

**Published:** 2026-04-17

**Authors:** Areti Dimitra Vrochari, Anastasia Agrafioti, Maria Dimitriadi, George Eliades

**Affiliations:** 1 Department of Endodontics, School of Dentistry, National and Kapodistrian University of Athens, 2 Thivon str, 11527 Athens, Greece; agrafiot@dent.uoa.gr; 2 Department of Biomaterials, School of Dentistry, National and Kapodistrian University of Athens, 2 Thivon Str, 11527 Athens, Greece; mardimit@dent.uoa.gr (M.D.); geliad@dent.uoa.gr (G.E.)

**Keywords:** calcium silicate-based endodontic sealers, setting time, hardness, solubility, bioactivity, reactivity with dentin, FTIR spectroscopy

## Abstract

The aim of this study was to evaluate setting time, hardness, solubility, bioactivity and interaction with dentin of four calcium silicate-based sealers (CSBS). Three single-phase CSBS (AH Plus Bioceramic/AHB, CeraSeal/CSL, TotalFill BC/TFL), one powder/liquid CSBS (BioRoot RCS/BRT) and an epoxy control (AH Plus Jet/AHP) were investigated. Setting time was evaluated on glass (G1) and dentin (G2) surfaces, by adding 1%wt purified water to single-phase products. For hardness measurements, the Shore-D hardness test was used. Solubility was assessed according to the ISO 6876:2012 standard. For bioactivity screening, 1-week set specimens were immersed in SBF or water (30 days/37 °C) and examined by ATR–FTIR spectroscopy. Interaction with dentin was tested by ATR–FTIR before and after contact with the sealers. For setting time in G1, all CSBS failed to comply with the ISO standard, while in G2, most materials were set in the range of 6–8 h, except for CSL. The ranking of significant differences in hardness was AHP, BRT > CSL, AHB, TFL. Regarding solubility, AHB, BRT and AHP were found to comply with the ISO standard, whereas CSL and TFL failed. For bioactivity, characteristic peaks of calcium phosphates were found in all CSBS, with TFL being the most bioactive. A chemical interaction between CSBS and dentin was registered, with a strong reduction in collagen peaks and an increase in carbonates. The CSBS tested exhibited great variance in their behaviour regarding the properties assessed, although a strong deproteinating effect was registered on dentin for all.

## 1. Introduction

Calcium silicate-based sealers (CSBS) have attracted the interest of researchers and clinicians due to evidence of enhanced biocompatibility [1,2,3,4,5] and increased bioactivity [2,4,6,7,8,9]. These sealers set via hydration and precipitation reactions. CSBS are available either in single-component premixed or in two-component formulations. For the first group, an external source of water is required for setting (i.e., intracanal moisture), while for the second, water is incorporated in the sealer during mixing prior to application.

Most CSBS produce film thickness within acceptable limits [5,9,10] and demonstrate increased flow [5,9,10,11,12,13,14]. Contradictory results have been reported for BioRoot RCS [10,11,13,14]. CSBS exhibit good sealing ability [15,16], with conflicting results on short- and long-term porosity [9,17,18]. This group of sealers shows very good antibacterial potential [1,3,5,19,20] mainly assigned to their high initial pH, without consensus on their pH stability over time [1,5,9,13,14,21].

The setting time of CSBS is one of the properties presenting conflicting evidence in the relevant literature. It has been shown that setting time is material-dependent. However, even among studies investigating the same product, different results can be found [5,9,11,22,23], depending on the methodology used. In clinical applications, the setting of single-component CSBS depends on the moisture found in root canal dentinal walls; therefore, it makes sense to involve dentin substrate in the research setup when setting time is investigated. Currently, such studies are scarce in the relevant literature [24].

Another controversial issue is the CSBS solubility. These sealers exhibit very high solubility values [1,3,5,9,12,14,21,25,26,27,28,29,30], although the opposite has been claimed, as well [10,23,31], with the storage medium being the principal regulating factor [5,8,23,,32].

The hardness of endodontic sealers has rarely been investigated. Hardness is extensively used in dentistry as an indicator of a material’s setting profile by monitoring resistance to indentation. A limited number of studies are available on CSBS hardness measurements [33,34,35,36].

Among the desirable properties of CSBS is their ability to induce hard tissue formation, due to their positive effect on osteogenic and cementogenic responses [2,3,4,6,37,38]. They also demonstrate increased Ca^2+^ ionic release when immersed in phosphate-buffer solution (PBS) or simulated body fluid (SBF) [5,39]. Finally, they induce precipitation of a mineral layer onto their surfaces when stored in PBS, SBF or Hank’s balanced salt solution (HBSS) [8,9]. In most cases, bioactivity is assessed by analysis (SEM/EDS, vibrational spectroscopies) of material surfaces before and after exposure to polyelectrolyte solutions. However, comparative data is limited.

Information on the morphological and structural effects of CSBS on dentin has received some attention, limiting the observations to the identification of a distinct interfacial zone with reduced porosity and an irregular pattern of sealer infiltration [40,41,42].

Therefore, the aim of the present study was to evaluate the setting time, solubility, hardness, bioactivity and interfacial properties with dentin of some modern CSBS. The null hypothesis was that there were no significant differences in the performance of the sealers.

## 2. Materials and Methods

Four CSBS and an epoxy control were selected for the study (Table 1). AHB, CSL and TFL are single-paste formulations, BRT is a powder/liquid formulation, and the control (AHP) is a paste/paste formulation.

### 2.1. Setting Time

Specimen discs (Ø: 19 mm, h: 1.5 mm, *n* = 3/sealer) were prepared, placed on microscopic glass slides and slightly overfilled with each sealer. For single-paste formulations (AHB, CSL, TFL), 1% wt reverse-osmosis purified water was added to accelerate setting, as advised by the ISO 6876:2012 standard for solubility testing [43]. For BRT, powder/liquid components were mixed as instructed, while for the paste/paste control (AHP), the material was directly applied in the moulds by the automix dispenser. The sealers were covered with a glass slip, pressed with finger pressure to remove excess, the slip was then removed by a sliding movement, and materials were left undisturbed in an incubator (98% RH/37 °C). After 5 h, the specimens were removed from the incubator, and a modified Gilmore-type straight indenter (80 μm diameter flat-end, 50 g mass) was used to assess the setting status. This procedure was repeated every hour until no indentation mark could be traced on the surface, and the time from mould application to setting was recorded.

An additional series of specimens (*n* = 3/sealer) was prepared by placing each sealer on blot-dried dentin discs prepared from a pool of extracted third molars of unknown origin, kept in 0.5% sodium azide solution at 5 °C (ΙRB: 745/27.01.2026). The crowns of the teeth were sectioned 1 mm above the pulp chambers level, and 1.5 mm apically with a diamond disc in a hard tissue microtome (Isomet 100, Buhler, Lake Bluff, IL, USA). The coronal surfaces were polished with 320–2000 grit size SiC papers in a grinding/polishing machine (Dap-V Struers, Bellarup, Denmark) and sonicated for 5 min in water to remove smears and blotted dry. Each sealer was applied in a thin layer using a micro-brush; no additional water was applied, and the samples were placed in the incubator. The setting time of the sealers was determined as previously described.

### 2.2. Hardness

Sealer specimens (Ø: 19 mm, h: 6 mm, *n* = 5/sealer) prepared as per manufacturers’ instructions (no additional water applied) were left undisturbed in an incubator (98% RH/37 °C) for 7 days to allow for full hydration and setting. Then, the specimens were stored in ambient conditions (45% RH/23 °C) for 1 week for maturation and subjected to hardness measurements. A Shore-D hardness tester was used (PCE-DD-D, PCE Instruments, Manchester, UK). Two measurements were obtained on each specimen plane surface, and the average was used as the representative specimen value.

### 2.3. Solubility

Specimen discs were prepared following the ISO 6876:2012 specification [43] for solubility testing. Plastic moulds (Ø: 19 mm, h: 1.5 mm, *n* = 3/sealer) were placed on microscopic glass slides. For AHB, CSL, and TFL, each mould was overfilled with the material, and 1% wt of reverse-osmosis purified water was added to accelerate setting. For BRT, powder/liquid components were mixed as advised, while for the paste/paste control (AHP), the material was directly applied in the moulds by the automix-dispenser. The overfilled moulds were covered with transparent plastic matrix strips and glass slides, and finger-pressed to remove excess. Then, the glass slides were removed by a sliding movement, and the moulds were placed in the incubator for 24 h. After setting, the specimens were demolded and finished with 800-grit SiC paper to remove flash and irregularities. The detailed procedure of solubility assessment according to the above specification is described in the Supplementary Material (S1).

### 2.4. Bioactivity

Disc-shaped sealer specimens were prepared as above (Ø: 19 mm, h: 2 mm, *n* = 15/sealer) and randomly divided into three groups per sealer (*n* = 5/group). The specimens of the first group were used as prepared (control), the specimens of the second group were immersed for 30 days/37 °C in distilled water, and the specimens of the third group were immersed under the same conditions in SBF (SBF, Biochemazone, Leduc, AB, Canada). The immersed specimens were then rinsed with distilled water, air-dried, and the exposed surfaces were examined by attenuated total reflectance Fourier transform infrared spectroscopy (ATR–FTIR). An ATR attachment (Golden Gate, Specac, Oprington, Kent, UK) was used with a 2 mm single reflection type III diamond reflective element, 45° ZnSe lenses and a sapphire anvil, attached to the sample compartment of an FTIR spectrometer (Spectrum GX, PerkinElmer, Buckinghamshire, UK). Spectra were acquired under the following conditions: 4000–650 cm^−1^ range, 4 cm^−1^ resolution, ≈2 μm depth of analysis at 1000 cm^−1^ and 20 scans coaddition. To semi-quantitatively assess the extent of bioactivity of the materials under the two storage conditions, the net area ratios of the complex PO_4_ peak (1180–970 cm^−1^) to the CO_3_ peak (1570–1410 cm^−1^) were calculated per specimen for each condition.

### 2.5. Interfacial Characteristics and Interaction with Dentin

Coronal dentin specimens prepared as previously described were covered with a thin layer of sealers. After setting (storage in 98% RH/37 °C for 7 days, *n* = 3/sealer), the specimens were embedded in fast-setting epoxy resin, sections were made to reveal the sealer-dentin interface and polished up to 1200 grit-size SiC papers. The exposed interfaces were examined under a reflected light microscope (DM 4000B, Leica, Wetzlar, Germany) and a SEM (Quanta 200, FEI, Hillsboro, OR, USA) operated in low-vacuum (LV) mode (0.13 MPa chamber pressure, 25 kV accelerating voltage, 90 μA beam current, without conductive coating) employing a solid-state backscattered electron detector for phase analysis (atomic number contrast).

Another series of dentin specimens (*n* = 3/sealer) prepared as above was used to evaluate the sealer interactions with dentin. ATR–FTIR spectra of the polished dentin surfaces were obtained under the conditions described in Section 2.4. The sealers were then applied to dentin. After 7-day storage in the incubator, the sealers were removed with sharp surgical blades until no sealer remnants were detectable under a stereomicroscope at 15× magnification (M80, Leica, Wetzlar, Germany). These surfaces were subjected again to ATR–FTIR analysis, and the resultant spectra were compared with their native dentin controls to identify changes in their chemical structure.

### 2.6. Statistical Analysis

The results of Shore-D hardness were analyzed by One-way ANOVA, while for the PO_4_/CO_3_ ratios, a 2-way ANOVA was used (independent factors: material type, storage condition). Post hoc multiple comparison tests were employed to identify group differences. All tests were performed using SigmaPlot v.15 software (SYSTAT Software Inc., Palo Alto, CA, USA) at a 95% confidence level (α = 0.05).

## 3. Results

### 3.1. Setting Time

According to the ISO standard, the setting time should be no more than 10% longer than the setting time claimed by the manufacturer, or it should be within the time range quoted by the manufacturer. The substrates used for testing were glass and dentin discs. The results of the setting time are presented in Table 2. On glass (G1), none of the CSBS tested complied with the specification (all were set after 24 h), except for the epoxy resin control (AHP). On dentin (G2), most materials set in a range of 6–8 h, apart from CSL, which exceeded 24 h. The control group was found to set earlier (6 h) than the setting time claimed by the manufacturer (8 h).

### 3.2. Hardness

The Shore-D hardness values of the sealers tested are shown in Table 3. Since the normality test failed (*p* < 0.05), a Kruskal–Wallis One-Way Analysis of Variance on Ranks and Dunn’s multiple comparisons tests were used. The ranking of the statistically significant differences was: AHP, BRT > CSL, AHB, TFL (*p* < 0.05).

### 3.3. Solubility

The results of the solubility according to the ISO standard are presented in Table 4. BRT, AHB and AHP were found to comply with the standard (solubility < 3.0%), whereas CSL and TFL failed, demonstrating solubility values higher than the 3.0% threshold.

### 3.4. Bioactivity

**Figure 1:**
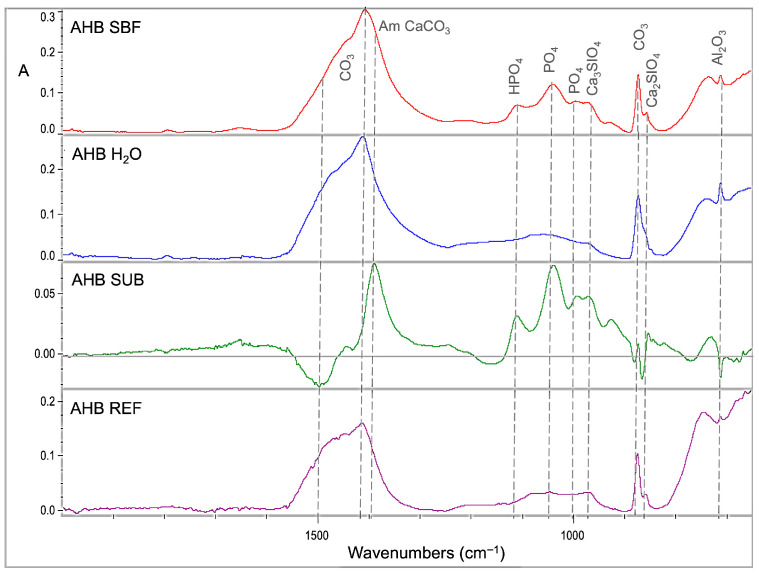
ATR–FTIR spectra of AHB after immersion in SBF (AHB SBF) and water (AHB H_2_O) for 30 days, along with the subtraction spectrum (SUB: AHB SBF–AHB H_2_O), the set non-immersed control (AHB REF) and the peak annotations (2000–650 cm^−1^ wavenumber range, A: absorbance scale).

**Figure 2:**
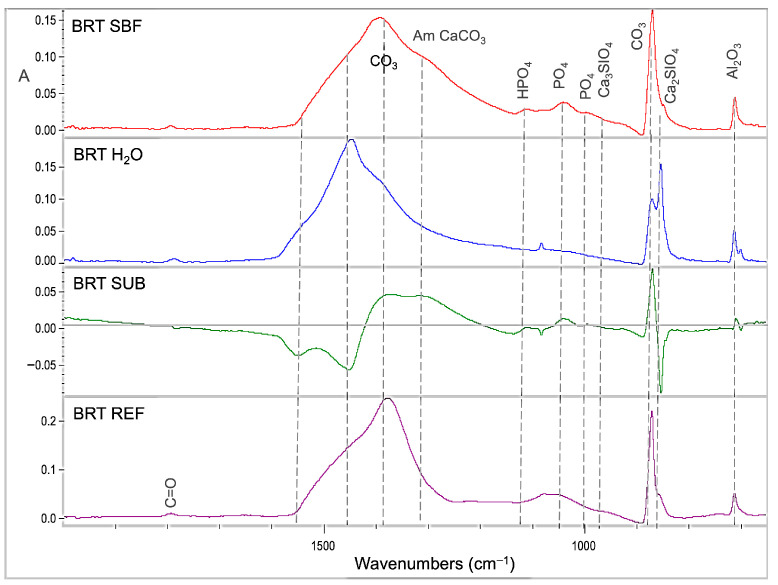
ATR–FTIR spectra of BRT after immersion in SBF (BRT SBF) and water (BRT H_2_O) for 30 days, along with the subtraction spectrum (SUB: BRT SBF–BRT H_2_O), the set non-immersed control (BRT REF) and the peak annotations (2000–650 cm^−1^ wavenumber range, A: absorbance scale).

**Figure 3:**
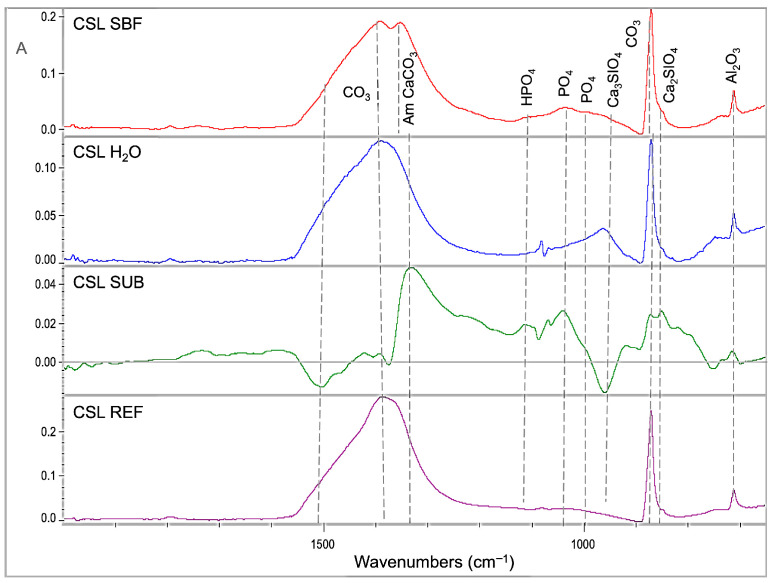
ATR–FTIR spectra of CSL after immersion in SBF (CSL SBF) and water (CSL H_2_O) for 30 days, along with the subtraction spectrum (SUB: CSL SBF–CSL H_2_O), the set non-immersed control (CSL REF) and the peak annotations (2000–650 cm^−1^ wavenumber range, A: absorbance scale).

**Figure 4:**
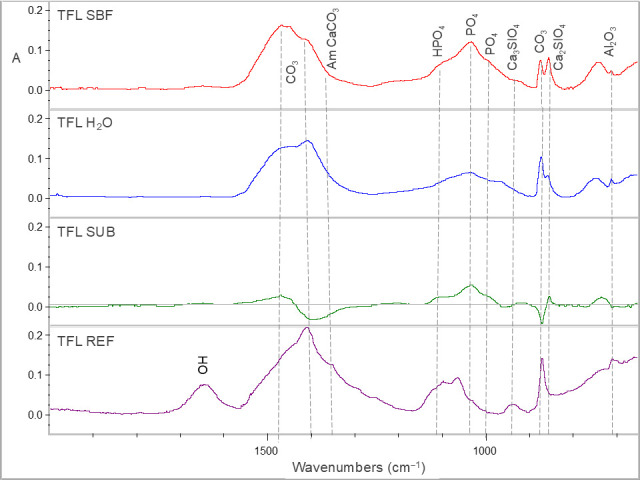
ATR–FTIR spectra of TFL after immersion in SBF (TFL SBF) and water (TFL H_2_O) for 30 days, along with the subtraction spectrum (SUB: TFL SBF–TFL H_2_O), the set non-immersed control (TFL REF) and the peak annotations (2000–650 cm^−1^ wavenumber range, A: absorbance scale).

**Figure 5:**
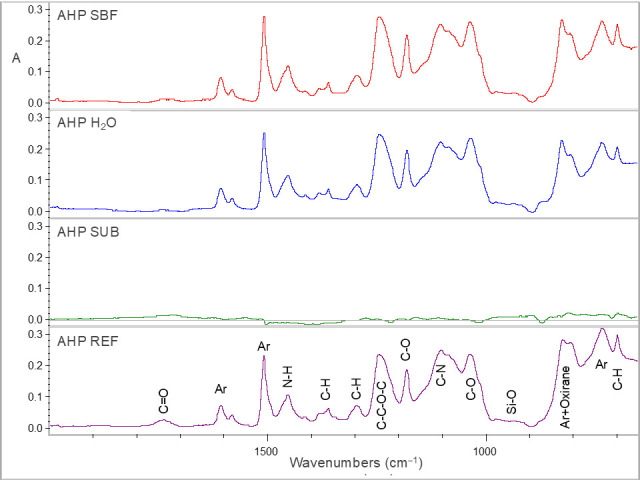
ATR–FTIR spectra of AHP (control) after immersion in SBF (AHP SBF) and water (AHP H_2_O) for 30 days, along with the subtraction spectrum (SUB: AHP SBF–AHP H_2_O), the set non-immersed control (AHP REF) with the peak annotations (2000–650 cm^−1^ wavenumber range, A: absorbance scale).

Representative sets of ATR–FTIR spectra, used for bioactivity assessment, are illustrated in Figure 1, Figure 2, Figure 3, Figure 4 and Figure 5. Each set includes sealer spectra (a) after storage in SBF, (b) after storage in water, (c) their difference (SUB: SBF–H_2_O), and (d) the set sealer before immersion. The peak assignments on each spectrum are according to the references presented in Table 5, as summarized in previous publications [44,45].

The spectra of AHB (Figure 1) showed an increase in phosphate, tricalcium silicate and amorphous carbonate peaks after SBF storage in comparison with water storage, which appeared more prominent in the difference spectra. These differences were also registered in comparison with SBF with the non-immersed control. On the other hand, water storage increased crystalline carbonate and dicalcium silicate peaks vs. the control.

The spectra of BRT (Figure 2) showed a minimal increase in phosphates and a moderate increase in amorphous carbonates after SBF storage, whereas water storage strongly increased crystalline carbonates and dicalcium silicates. The increase in dicalcium silicates after water storage was much higher than that of the control. The control exhibited less amorphous carbonate from immersed specimens, regardless of the immersion solution used, and higher absorbance at the phosphate and tricalcium silicate bands from the specimens stored in water.

For CSL (Figure 3), SBF storage increased the amorphous carbonates, phosphates and dicalcium silicates in comparison with water storage, but to a lesser extent. Water storage increased the absorbance of crystalline carbonates and tricalcium silicates. In comparison with the non-immersed control, SBF increased the peaks of amorphous carbonates, phosphates, and tricalcium silicates, whereas water increased the crystalline carbonates and tricalcium silicates, the latter to a greater extent than SBF.

For TFL (Figure 4), SBF increased crystalline carbonates, phosphates and dicalcium silicates in comparison with water, while the latter increased the partially amorphous carbonates. The non-immersed material showed a typical –OH peak assigned to Ca(OH)_2_, partially amorphous carbonates and acidic phosphates (HPO_4_) in comparison with all immersed specimens.

Spectra of AHP (control) showed no changes in any state, indicating a lack of bioactivity after H_2_O or SBF immersion (Figure 5).

The results of the PO_4_/CO_3_ peak area ratios after CSBS storage in H_2_O and SBF are presented in Table 6.

Data passed normality (Shapiro–Wilk test, *p* = 0.319) and equal variance tests (Brown-Forsythe test, *p* = 0.179). The 2-way ANOVA (independent factors: material type and storage conditions) showed a significant interaction between the levels of the two factors (*p* < 0.001). Pairwise multiple comparison tests (Holm–Sidak test) demonstrated significantly higher PO_4_/CO_3_ ratio after SBF storage (*p* < 0.05). The ranking of the significant differences within each group was (*p* < 0.05): TFL > AHB, CSL, BRT (for H_2_O) and TFL > AHB > CSL, BRT (for SBF).

### 3.5. Interfacial Characteristics and Interaction with Dentin

**Figure 6:**
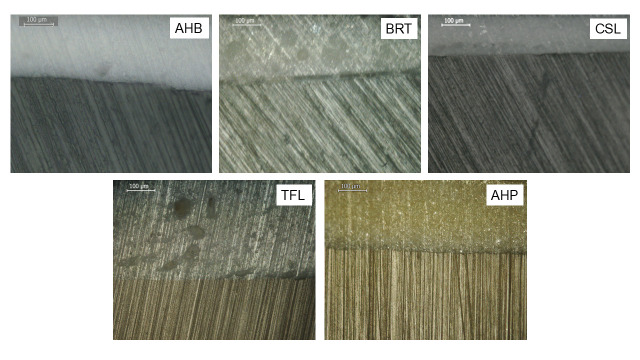
Reflected-light microscopic images of the material–dentin interface (100× magnification, bar: 100 μm).

Reflected light microscopic images of the dentin-sealer interfaces are illustrated in Figure 6. The resinous AHP control showed a continuous, porous-free interface with dentin. All CSBS materials demonstrated interfacial and bulk material defects. BRT and TFL showed interfacial debonding, and showed AHB, CSL, and TFL porosity in bulk material and at the interface.

**Figure 7:**
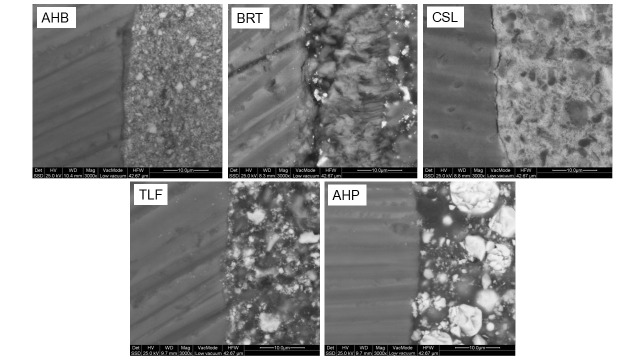
Backscattered LV–SEM images of the material-dentin interfaces (3000× magnification, bar: 10 μm).

The images of the same interfaces observed at higher magnification under the LV–SEM employing a backscattered detector are illustrated in Figure 7. Again, the AHP control exhibited the best interfacial quality, with a low atomic number contrast continuous resinous phase in contact with dentin. AHB, BRT and CSL demonstrated defects and extensive interfacial debonding, whereas TFL showed minor debonding at the interface.

**Figure 8:**
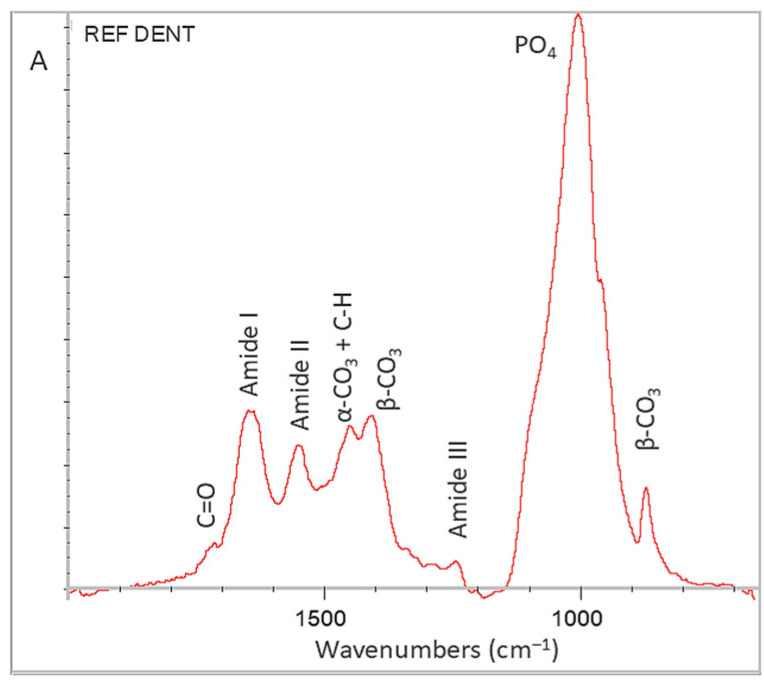
ATR–FTIR spectrum of reference dentin used as substrate for sealer bonding (2000–650 cm^−1^ range, A: absorbance scale).

A representative ATR–FTIR spectrum of dentin specimen (2000–650 cm^−1^ range) used for sealer applications is presented in Figure 8. The typical peaks identified were (cm^−1^): 1720 (C=O str of COOH assigned to fragmented collagen peptides), 1650 (C=O str of RCONHR-amide I), 1540 (N–H b + C–N str of RCONHR-amide II), 1450 (C–H str + C–H b overlapping with *α*–CO_3_), 1405 and 870 (*β*–CO_3_), 1250 (C–N str + N–H b of RCONHR-amide III) and 1185–885 (the complex peak of calcium phosphates including contributions of HPO_3_, PO_4_ amorphous and crystalline) [56].

**Figure 9:**
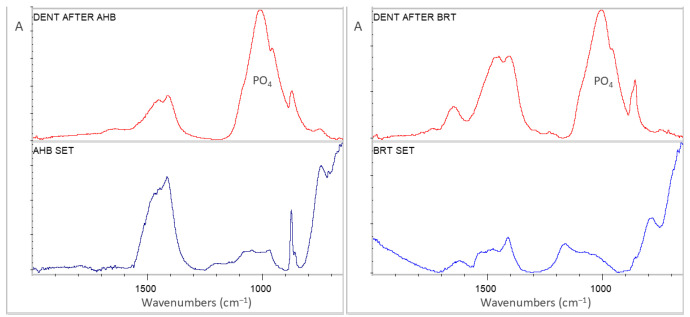
ATR–FTIR spectra of dentin specimens after removal of AHB (**left**) and BRT (**right**) materials, along with the corresponding material spectra (2000–650 cm^−1^ range, A: absorbance scale).

**Figure 10:**
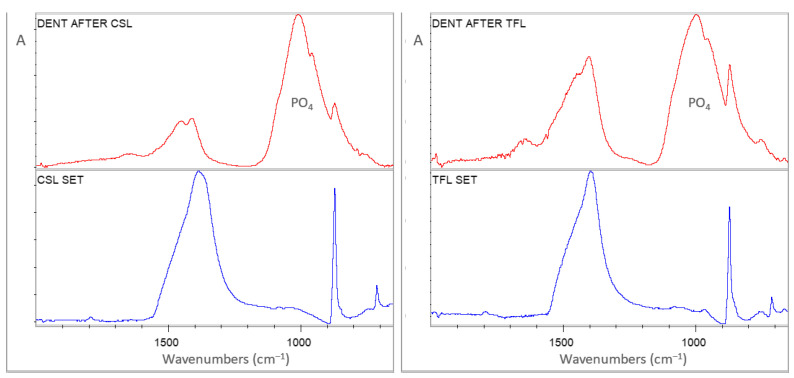
ATR–FTIR spectra of dentin specimens after removal of CSL (**left**) and TFL (**right**) materials, along with the corresponding material spectra (2000–650 cm^−1^ range, A: absorbance scale).

Figure 9 and Figure 10 demonstrate ATR–FTIR spectra of two representative dentin specimens per CSBS sealer, after removal of the sealer, along with the spectrum of the set material. All spectra of treated dentin specimens resembled that of reference dentin, with the following major differences: first, the collagen peaks (amide I, II and III) were diminished in all specimens, and second, the carbonate peaks were increased. The small peaks at 1650–1640 cm^−1^ of treated dentin spectra are mostly assigned to –OH groups, and not to amide I, since no amide III peaks were traced at 1250 cm^−1^, where the sealer interferences are minimal. The presence of the strong apatite-derived phosphate peak implies that the main dentin surface was accessible without material interferences. It was not possible to apply the same type of analysis on dentin specimens treated with the control AHP, since the resin strongly adhered to the substrate.

## 4. Discussion

The setting reaction of CSBS is complex and integrated into many steps. However, the clinically relevant solid phase transformation is determined by the setting time. According to the ISO 6876:2012 specification for setting time [43], the preparation of single-paste CSBS, which requires moisture for setting, should be made by the “moist method” in hydrated type II gypsum moulds. Yet, it is not known whether the amount of water the sealers absorb during this procedure is less, adequate or more for completion of the setting reaction. In the currently available literature, water was added in specific amounts to determine how CSBS setting time is affected in vitro [22], while Wistar rats were employed in an animal study to reveal the effective sealer setting times [57]. During pilot tests of the present study, it was noticed that CSBS with a prolonged setting time (>72 h) set faster (24–48 h) when mixed with 1% wt of water, prior to placement in plastic ring moulds on a glass substrate. Therefore, for setting time, this modification was followed, which complies with the ISO 6876:2012 specification for solubility testing [43], where a major prerequisite is proper material setting. Consequently, the addition of 1% wt seems a more predictable method of measuring setting time than the “moist method”. With this setup, the setting times recorded were close (AHB, 24 vs. 20 h) or the same (TFL) with gypsum moulds [5]. Overall, there are great discrepancies in the methodology used in the relevant literature, since some studies demonstrated faster setting with the “moist” method [58], while others failed to detect setting even after 25 days of storage, both for the “moist” and “dry” method of specimen preparation [57]. It seems that the determining factor for setting time is the chemical composition of the CSBS, which greatly varies among the single-paste materials used. BRT, distributed in a powder/liquid formulation, does not require the addition of external water to set, thus reducing the inherent water sensitivity of single-phase systems. Nevertheless, in the present study, longer setting times have been registered than in some previous studies [11,23,59], possibly assigned to differences in the consistency of the components (i.e., packed or loose powder, thinner or thicker liquid drops, etc).

The second part of the setting time study was performed by applying the sealers on dentin discs instead of glass, conditions being closer to the clinical application. The setting time of all CSBS was expedited when in contact with dentin. AHP and BRT demonstrated setting times close to the manufacturers’ information, while for AHB and TFL, the setting time was decreased to 8 h, a clinically reasonable period in comparison with the AHP control. CSL showed the longest setting time on dentin (24 h). A possible explanation for setting acceleration on dentin is the amount of loosely bound water of the tissue [22]. Moreover, the very high pH of these sealers may further absorb a fraction of the chemisorbed dentin water, introducing a “burning effect” on dentin collagen [60]. So far, there are only a couple of studies where dentin has been used as a substrate for setting time assessment. In an animal study [57], the positive effect of dentin on TFL setting was confirmed, although the first assessment interval was after 1 week, while in an in vitro study, moist dentin accelerated CSBS setting [24]. These findings may raise questions as to whether some of the testing specifications for these materials need to be revised, using more standardized conditions and clinically relevant substrates. At the stage of preparation of the present manuscript, a new version of the ISO specification (ISO 6876:2025-11, released on 5 November 2025) was introduced [61], where the “moist method” was abandoned, and the use of metallic or plastic moulds is advised, without any other source of water than exposure to ≥95% RH. The revised version simplifies the procedure and may provide more relevant results. However, since the setting of the materials depends on externally diffused water, the dentin model seems most reliable, regardless of the biological variance of the tissue.

For hardness testing, no external water was added to the specimens. Instead, based on pilot tests, the specimens were left undisturbed in the incubator (98% RH/37 °C, 7 days) for full hydration and setting, and 1 week at room temperature for further maturation. The hardness of endodontic sealers has been rarely investigated, as it has been claimed that it lacks direct clinical significance. However, it may offer important information for the material’s resistance to indentation and cohesive strength build-up rate during the setting process. For this purpose, it has been extensively used in testing a wide range of dental materials. Moreover, hardness is related to abrasion resistance [62] and therefore may be implicated in the retreating capacity of root canals sealed with CSBS, since very hard materials are difficult to remove with rotary instruments [63]. The Shore-D method was employed for testing, as CSBS materials are quite brittle. The two-component BRT and the AHP (control) demonstrated the highest hardness values among the materials tested. The greatest hardness of BRT from TFL and AHB has already been documented in a recent study [64] and has been mainly assigned to the lower calcium silicate content of AHB.

Solubility of CSBS has generally been reported to be relatively high, due to their hydrophilic nature and strongly dependent on the type of immersion media [5,8,21,23,29,56]. In the present study, the instructions given in the ISO 6876:2012 standard were followed to assess the short-term solubility in water. The results showed that AHB, BRT and AHP (control) comply with the ISO specification, contrary to other studies [1,8,9,14,21,26,28]. However, the methodology used in these studies demonstrated deviations from the ISO standard in terms of specimen preparation and assessment method. TFL and CSL failed to comply with the ISO specification in agreement with previous studies [1,3,12,21,25,27,28,29], although compliance was presented in one study for CSL [9], but with a different methodology. The two-component system BRT fulfilled the ISO requirements, in agreement with previous studies [23]. The differences in the solubility within the CSBS materials tested should be assigned to variations in the calcium silicate content. AHB contains 5–15% wt tricalcium silicate, which is much lower than the amount of tricalcium silicate (20–30% wt) and dicalcium silicate (1–10% wt) of CSL and tricalcium silicate (20–35% wt) plus dicalcium silicate (7–15% wt) of TFL, according to the manufacturers’ information. As calcium silicates are the hydrophilic components of these materials, the lower content of AHB might explain the lower solubility values. It should be mentioned that the AHB sealer has been withdrawn from the market and replaced by a new sealer (late 2025). Nevertheless, the performance of the material is still the subject of recently published studies [65,66,67,68,69,70,71], which may facilitate future developments in the field regarding structure-property relationships of CSBS. BRT, on the other hand, does not provide information about the tricalcium silicate content. This sealer does not require external moisture to set, as it comes in a liquid/powder formulation. The two-component predefined BRT composition, with the addition of calcium chloride as an accelerating agent and polycarboxylate salt in the liquid component, may explain the more predictable setting performance of this material. In the latest version of the ISO standard (ISO 6827: 2025-11) [61], the testing procedure has been revised by allowing the materials to set in an incubator for 7 days, as in the hardness testing proposed above. Nevertheless, since the materials tested were officially evaluated for compliance with the ISO 6827:2012, comparisons were based on this version. The importance of CSBS solubility has been a matter of debate in relevant literature. It has been claimed that solubility testing requiring external water to set is problematic, suggesting dimensional stability assessment as more appropriate. Within this context, it has been demonstrated that CSBS possesses very high solubility values, exhibiting dimensional stability similar to the control when examined via micro-XCT [12,29]. Solubility is also related to substance release and ionic leaching (i.e., Ca(OH)_2_, Ca^2+^), on which CSBS relies to enhance its biological properties. Therefore, some authors question the importance of low solubility values for this type of material. However, high solubility values may transform a permanent sealer into an intermediate long-term intracanal dressing, deviating from the major principles of root canal sealing. Considering this critical point, the setting time, hardness and solubility are of paramount importance to establish structure-property relationships for CSBS, which merit further investigation.

The bioactivity of CSBS has been considered a major advantage. Root canal sealers come into direct contact with the periapical tissues through the apex during the obturation process. Thus, sealers with increased bioactivity may potentially lead to enhanced healing [72]. Also, increased mineralization potential may result in better adaptation of the material to the canal walls and apex [72,73], provided that the materials have low solubility. Bioactivity is mainly tested by investigating the capacity of sealers to induce hydroxyapatite precipitation on their surfaces after exposure to PBS, SBF or HBSS solution [8,9]. ATR–FTIR spectrometry is a powerful atmospheric and non-destructive surface analysis method for bioactivity assessment by directly tracing the presence of phosphates and carbonates, assigned to hydroxyapatite and its precursors [8]. In the current study, an increase in the phosphate and carbonate peaks was observed after SBF immersion compared to water immersion. In addition, some differences in the bioactivity pattern were noticed between the materials tested. For example, when TFL was immersed in SBF, crystalline carbonate peaks increased, contrary to the other sealers that presented an increase in amorphous carbonates. In water, TFL exhibited partially amorphous carbonates, while the rest of the sealers showed crystalline carbonates. Moreover, the non-immersed TFL showed a greater extent of –OH vibrations (possibly assigned to Ca(OH)_2_), partially amorphous carbonates and acidic phosphates (HPO_4_) in comparison with all immersed specimens. These differences should be attributed to the variations in the calcium tri-/di-silicate contents, which may affect the amount of Ca(OH)_2_ produced and the resultant reaction pattern with the immersion media. In the present study, the PO_4_/CO_3_ ratio has been used as an indication of bioactivity. The ratio was significantly increased after SBF immersion, which implies that (a) PO_4_ and CO_3_ peaks exist on CSBC surfaces after water exposure, and (b) the PO_4_ groups are significantly increased after SBF exposure, apparently due to the development of calcium phosphate precipitates. This agrees with previous studies where the P and Ca constituents of original CSBS increase in content after water storage [44], and especially after immersion in PBS or SBF solutions [8,9,41]. The highest PO_4_/CO_3_ ratio of TFL should be attributed to the highest content of tri- and di-calcium silicates.

An important question for CSBS concerns their interfacial reactivity with dentin. In the current study, the dentin–sealer interface was examined without any means of ageing (PBS or SBF) other than a 7-day storage period in an incubator for proper material setting. A distinct interface between all sealers and dentin was observed, with some defects (cracks, pores) observed in CSBS. At higher magnification, under the LV–SEM conditions to avoid excessive specimen dehydration and the resultant artifacts, no infiltration tags could be seen. On the other hand, tag-like structures have been observed at the interface of a CSBS after 2 weeks of storage in PBS [41]. These materials create a mineralized interfacial zone in adjacent dentin due to caustic degradation of collagen by the high pH of the silicate sealers. The zone has been reported to be rich in carbonates, without significant changes in the amide I to phosphate peak ratios of dentin [60,74]. In the present study, the major peaks of dentin collagen (amide I, II and III) were strongly reduced, along with an increase in carbonates after contact with the CSBS. The increase in carbonates agrees with previous studies [60,74]. However, in the present study, the amide peaks were strongly reduced, contrary to previous findings [60], which show a great extent of collagen degradation. Moreover, the strong phosphate peak of dentin hydroxyapatite structure confirms the minimal extent of original sealer interferences within the probed depth (superficial ≈2 μm). The spectra of intact dentin after CSBS treatment resemble those of the NaOCl-treated intact dentin regarding the decrease in amide and increase in carbonate groups [75], with a stronger reduction in amide groups in the former due to the increased exposure time and the highest pH of CSBS. It is not known if this interaction may induce dentin mineralization after prolonged exposure. It should be pointed out, though, that no adhesion of calcium silicate sealers was evident, as the materials could be scraped off the dentin surface quite easily. The interfacial reactions of high-pH sealers with dentin should be carefully revised and critically approached. The reaction of these materials seems similar to calcium hydroxide, but probably more extended, for which important limitations have been reported associated with reduced root mechanical properties [76,77]. Nonetheless, preliminary data for one sealer showed negligible changes in dentin mineralization and crystallinity in comparison to the severe changes observed for a calcium hydroxide sealer [74]. Undoubtedly, the interaction of the new sealers with dentin should be studied in more detail, considering the various treatment modes of exposed dentin during chemomechanical root canal preparation.

The clinical relevance of the in vitro tests employed in CSBS should be critically assessed. Setting time may express the limit required before safe application of restorative materials (i.e., acidic conditioners, adhesives, liners, post space preparation and placement, etc), on a high pH CSBS substrate, without affecting the procedures. Moreover, a very extensive setting time may increase the risk of coronal leakage, mainly through temporary non-adhesive filling materials. Solubility is a critical property for the long-term performance of the entire root-canal filling process, since failure may occur due to intraoral fluid and microbial leakage, which may affect the integrity of the sealer and the sealer-dentin interface [78]. Hardness, although underestimated in the field, may provide important information about the sealer setting profile, post-setting reactions, environmental plasticization effects and abrasion resistance of the sealers associated with retrieval capacity [63]. Bioactivity, apart from repair of periapical tissues, may assist sealer solubility, since crystalline hydroxyapatite formation increases acid-resistance, contributes to dentin mineralization and enhances interfacial bonding with dentin [79].

The results of the present study showed important differences in the performance of the CSBS materials tested, with great variances in the results obtained among the CSBS sealers or in comparison with the control. Therefore, the null hypothesis should be rejected, except for CSL, AHB and TFL comparisons in hardness. Moreover, several tests revealed the need for more precise and efficient standardization procedures, since there are huge deviations in the reported results.

## 5. Conclusions

The calcium silicate-based sealers tested exhibited different behaviour to the control epoxy sealer in most properties assessed. Moreover, and possibly more importantly, major differences were found in the properties tested within the group of calcium-silicate-based sealers, which may affect their clinical performance.

## Figures and Tables

**Table 1:** The endodontic sealers used in the study.

Sealer/Lot	Code	Composition *	Manufacturer
AH Plus Bioceramic (KI210816)	AHB	Paste: Tricalcium silicate (5–15% wt), Zirconium dioxide, Dimethyl sulphide, Lithium carbonate, thickening agent.	Dentsply Sirona, Charlotte, NC, USA
BioRoot RCS (B27246)	BRT	Powder: Tricalcium silicate, Zirconium oxide, Povidone hydrophilic biocompatible polymer. Liquid: Water, Calcium chloride, Polycarboxylate (water-reducing agent).	Septodont, St. Maur-des-Fossés, France
CeraSeal (CSL2112092)	CSL	Paste: Tricalcium silicate (20–30% wt), Dicalcium silicate (1–10% wt), Calcium aluminate, Zirconium oxide, thickening agent.	Meta Biomed Europe, Műlheim an der Ruhr, Germany
TotalFill BC (21004SP)	TFL	Paste: Tricalcium silicate (20–35% wt), Dicalcium silicate (7–15% wt), Calcium phosphate monobasic, Zirconium oxide, Tantalum oxide, Calcium hydroxide, filler and thickening agents.	FKG Dentaire, La Chaux des Fonds, Switzerland
AH Plus Jet (2110001038)	AHP	Epoxide paste: Diepoxide, Calcium tungstate, Zirconium oxide, Iron oxide, pigment. Amine paste: 1-Adamantane amine, N, N’ Dibenzyl-5-oxa-nonandiamin-1,9, TCD-diamine, Calcium tungstate, Zirconium oxide, Aerosil, and Silicon oil.	Dentsply Sirona, Charlotte, NC, USA

* According to the manufacturers’ information.

**Table 2:** The results of the setting time.

Sealer	Setting Time on Glass (G1/h)	ISO Status (<+10%)	Setting Time on Dentin (G2/h)
AHB	>24	Fail	6
BRT	>24	Fail	6
CSL	>24	Fail	24
TFL	>24	Fail	8
AHP (Control)	6	Pass	6

**Table 3:** The Shore-D hardness values of the materials tested (medians and interquartile ranges) *.

Sealer	Shore-D Hardness
AHB	55.4 (49–58) ^b^
BRT	74.5 (70.7–78.33) ^a^
CSL	59 (54.8–59.8) ^b^
TFL	54.6 (50.7–55.9) ^b^
AHP (Control)	75.5 (72.5–77.3) ^a^

* Same letters indicate median values with statistically insignificant difference (*p* > 0.05).

**Table 4:** The results of solubility according to ISO 6876:2012.

Sealer	ΔW (g)	3% ΔW ISO Acceptance Limit (g)	ISO Status (<3%)
AHB	0.02	0.033	Pass
BRT	0.030	0.069	Pass
CSL	0.062	0.032	Fail
TFL	0.049	0.031	Fail
AHP (Control)	0.011	0.040	Pass

**Table 5:** Assignment of ATR–FTIR peaks of CSBS.

Wavenumber (cm^−1^)	Chemical Group	References
1455	–CO_3_ (*v1*)	[46]
1420	Amorphous –CO_3_ (*v1*)	[47,48]
875	–CO_3_ (*v2*)	[46,47]
1065–1000	–PO_4_ (*v2*, *v3*)	[49,50,51,52]
665	–PO_4_ (*v4*)	[50]
936	–Si–O of Ca_3_–silicates	[46,53]
850	–Si–O of Ca_2_–silicates	[47,53,54]
743	Al–O of Al_2_O_3_	[55]

**Table 6:** The results of PO_4_/CO_3_ peak area ratios of the CSBS after H_2_O and SBF storage (means and standard deviations) *.

Sealer	PO_4_/CO_3_ Ratio	
	H_2_O Storage	SBF Storage
AHB	0.05 (0.02) ^A,a^	0.14 (0.02) ^B,a^
BRT	0.03 (0.01) ^A,a^	0.08 (0.01) ^B,b^
CSL	0.05 (0.01) ^A,a^	0.09 (0.01) ^B,b^
TFL	0.18 (0.03) ^A,b^	0.56 (0.03) ^B,c^

* The same upper-case letters show insignificant differences between storage conditions per material, while lower-case letters show insignificant differences among materials per storage condition (*p* < 0.05).

## Data Availability

The original contributions presented in this study are included in the article/Supplementary Material. Further inquiries can be directed to the corresponding authors.

## Supplementary Materials

The following supporting information can be downloaded at: https://www.mdpi.com/article/10.3390/jfb17040192/s1, S1: Solubility assessment according to ISO 6876:2012.
