# Supplementary material for: Setting Characteristics, Solubility, Bioactivity and Interaction with Dentin of Four Calcium Silicate-Based Endodontic Sealers
Source: J Funct Biomater. 2026 Apr 17;17(4):192. doi: 10.3390/jfb17040192 (PMC13117805; doi:10.3390/jfb17040192)
Supplement: Supplementary file 1 [file jfb-17-00192-s001.zip › jfb-4218681-supplementary.pdf]

Supplementary material:

S1: Solubility assessment according to ISO 6876:2012.

The final material mass of each specimen was weighed (± 1 mg) in an analytical balance. Two specimens of each material were placed in a shallow porcelain dish A (Ø: 90 mm, minimum volume: 90 ml) without being in contact, 50 ml of water were added, the dish was covered and placed in the incubator for 24 h. A fluted filter was prepared and placed into a funnel located 20 mm above the bottom of a pre-weighed swallow porcelain dish B. After 24 h, the water together with the specimens were poured into the fluted filter. Dish A was rinsed three times with 5 ml of water, which was also poured into the fluted filter. Dish B, along with the collected water, was placed in an oven at 110°C and left until a constant mass was obtained with the dish cooled in the desiccator to room temperature. The difference between the original and final mass of dish B was recorded as the amount of dissolved sealer. The difference in mass was expressed as a percentage of the original combined mass of the two specimens, to the nearest 0.1%. The test was carried out twice, as prescribed by the ISO standard and the mean value was recorded as the sealer solubility.
